# A Simple and Efficient Method for Finger Vein Recognition

**DOI:** 10.3390/s22062234

**Published:** 2022-03-14

**Authors:** Zhongxia Zhang, Mingwen Wang

**Affiliations:** School of Mathematics, Southwest Jiaotong University, Chengdu 611756, China; zhangzx@my.swjtu.edu.cn

**Keywords:** finger vein recognition, centrosymmetric coding, minimum Hamming distance, generating code

## Abstract

Finger vein recognition has drawn increasing attention as one of the most popular and promising biometrics due to its high distinguishing ability, security, and non-invasive procedure. The main idea of traditional schemes is to directly extract features from finger vein images and then compare features to find the best match. However, the features extracted from images contain much redundant data, while the features extracted from patterns are greatly influenced by image segmentation methods. To tackle these problems, this paper proposes a new finger vein recognition algorithm by generating code. The proposed method does not require an image segmentation algorithm, is simple to calculate, and has a small amount of data. Firstly, the finger vein images were divided into blocks to calculate the mean value. Then, the centrosymmetric coding was performed using the matrix generated by blocking and averaging. The obtained codewords were concatenated as the feature codewords of the image. The similarity between vein codes is measured by the ratio of minimum Hamming distance to codeword length. Extensive experiments on two public finger vein databases verify the effectiveness of the proposed method. The results indicate that our method outperforms the state-of-the-art methods and has competitive potential in performing the matching task.

## 1. Introduction

Finger vein recognition has emerged from a fairly new topic a few years ago to significant deployed systems and has demonstrated a reasonably good recognition performance [1,2]. It can capture the texture features under the blood vessels from different viewpoints such as palm side [3], dorsal side [4], and periphery of the finger [5]. Compared with other biometric technologies, a finger vein image has the following advantages for personal authentication: (1) Safety: vein pattern is an internal feature and not easy to replicate [6]; (2) Living body identification: only the vein in a living finger can be captured and further used in identification [7]; (3) Non-contact: the aging and wear of the skin surface can be ignored because finger veins are located in the subcutaneous layer of the skin.

In recent years, a variety of methods have been proposed for finger vein recognition, which can be roughly divided into the following categories according to the different methods of feature extraction.

### 1.1. Vein Pattern Methods

The method based on vein pattern is to segment vein pattern from finger vein image, and match vein pattern by geometric shape or topological structure. Typical methods include repeated linear tracking, RLT [8], maximum curvature, MCP [9], mean curvature, MC [10,11], Gabor [12], etc. Recently, some improvements have been made to robust vein pattern extraction. Yang et al. [13] proposed a finger vein code index method, and combined it with the finger vein pattern matching method into an integrated framework to improve accuracy and efficiency. Experimental results indicate that the integration framework highly improves the identification efficiency with a slight improvement on the accuracy. However, due to environmental and other factors, only a small number of veins may be captured in the finger vein images, or a large number of veins may be captured but with irregular shadows and noise. For such images, it is difficult to avoid oversegmentation of the vein pattern or undersegmentation of the vein pattern, which makes it a challenge to represent finger vein images effectively. Based on this, the authors in [14] used a low-rank representation to extract as much noise-free discriminative information from finger vein images for more effective and robust finger vein recognition. This scheme can extract more important information from low-quality images.

### 1.2. Feature Points Matching Methods

The method based on feature points matching is where matching is performed by detecting minutiae points of the image, or other types of feature points. Minutiae includes bifurcation points and end points. Typical methods based on minutiae include minutiae matching based on improved Hausdorff distance matching [15] and minutiae matching based on singular value decomposition [16]. The minutiae-based method needs to segment the finger vein similar to in the vein-pattern-based method, and then extract the minutiae from the texture. In finger vein image, the number of minutiae is small, which is a problem in the application of minutiae-based method to the recognition task of finger vein image. In [17], a zone-based minutia matching technique, which combines minutia matching with traditional region-of-interest (ROI)-based method, was designed to deal with these problems. The author selected the minutiae in a rational neighborhood zone for matching, abandoning unnecessary matching, and avoiding false matching to some extent. In addition, the SIFT [18,19] method can extract more feature points from finger vein images. However, the fuzzy vein patterns of finger vein images can easily lead to false detection of feature points, and the deformation of vein lines caused by finger bending or rotation is not considered. Matsuda et al. [20] proposed a new feature-point-based matching method by using the curvature of the image-intensity profiles to extract feature points, which is more robust to both irregular shadows and texture distortion, and obtained higher matching accuracy than the SIFT method. However, the scheme has high time cost and needs to consider the normalization of image angle and scale in practical applications.

### 1.3. Statistical Characteristic Analysis Methods

The basic principle of statistical-characteristics-analysis-based schemes, such as principal component analysis (PCA) [21,22], linear discriminant analysis (LDA) [23], and sparse representation (SR) [24], does not need to extract finger vein lines, but uses all image information (including vein region and non-vein region) for identification. Wang et al. [21] used PCA to reduce the dimensionality of the image to obtain the main components of the finger vein image. However, this method does not consider the supervision information, and when the sample size is large, the time complexity is high. On the basis of PCA dimension reduction, Wu and Liu [23] proposed LDA to further reduce dimension and extract distinguishing features. This method takes into account the supervision information, but it is difficult to calculate when the amount of data is large and the dimensionality is too high. Xin et al. [24] successfully applied SR to finger vein recognition tasks. Furthermore, Li et al. [25] used sparse feature descriptors to adaptively project directional difference vectors into a feature space with discriminative binary codes to better represent the directional features of finger vein images, increasing the distance of inter-instance samples while reducing the distance of intra-instance samples. The above methods can reduce the preprocessing steps and have small space occupation of feature vectors. However, these methods take the whole image as data, learn the overall structure of all images, and cannot sufficiently consider the local detail features of the image, which has a detrimental influence on the accuracy of finger vein recognition.

### 1.4. Local Features Methods

The method based on local features, which also does not need to segment the image, is widely used in finger vein recognition [26,27]. These methods include local binary mode (LBP) [28] and local derivative mode (LDP) [29]. Many LBP variants have also been proposed [30]. Zhang et al. [31] presented directional binary code, which is a new LBP variant. Yang et al. [32] suggested to use LBP-based personalized best bit mapping. Experimental results show that the method not only has better accuracy, but also has higher reliability and robustness. Recently, Petpon et al. [33] came up with a new LBP variant called local line binary pattern (LLBP), and soon Rossi et al. [34] applied it in finger vein recognition. Its accuracy was better than that of LBP and LDP, and it was applied to near-infrared face recognition. The traditional local binary feature extraction method extracts features from each pixel point in the image, which has a large number of features and contains redundant information, and the overextraction process does not perform the dimensionality reduction operation. For the LBP dimensionality problem, Li et al. [35] proposed the partitioned local binary pattern (PLBP) algorithm for dorsal hand vein recognition, and the choice of partition size has a great impact on the recognition rate. The higher the number of partitions, the higher the recognition rate. When the number reaches a certain level, the recognition rate does not decrease. Liao et al. [36] used multi-scale block local binary pattern (MB-LBP) for face recognition. The MB-LBP operator encodes not only the microstructure of the image pattern but also the macrostructure, providing a more complete image representation than the basic LBP operator. In addition, the centrosymmetric local binary pattern (CS-LBP) was proposed in [37], which has only 1/8 of the feature dimension of LBP and is also faster to process than LBP. However, CS-LBP analyzes texture features from a microscopic perspective, ignoring the larger texture structure features, and few studies use CS-LBP directly.

### 1.5. Deep Learning Methods

In the field of finger vein verification, the deep-learning (DL)-based approach has been successfully applied in recent years [38]. It consists of a deep neural network (DNN), which can provide powerful image processing capabilities without any prior knowledge [39], and has good adaptive performance in noise image processing and feature representation learning [40]. For example, a deep convolutional neural network (CNN) with five convolution layers and two fully-connected layers was proposed to design a new finger vein recognition method, which is able to achieve better performance than traditional algorithm [41]. The multi-receptive field bilinear convolutional neural network (MRFBCNN) network designed in [42] can obtain the second-order features of finger veins and better distinguish finger veins with small differences between classifications. Moreover, a lightweight neural network is used to reduce the network parameters and computational complexity. Fairuz et al. [43] developed a finger vein identification system using transfer learning of alexnet model and tested it with receiver operating characteristic curve (ROC) curves to analyze the outcomes of the experiments, with satisfactory results. Convolutional neural networks have been proven to have strong feature representation ability. However, they often require large training samples and high computation that are infeasible for real-time finger vein verification. To address this limitation, Fang et al. [44] proposed a lightweight DL framework for finger vein verification, and proved that the two-channel network can be trained by increasing the training sample through an exquisite topological structure. However, the proposed system is not yet perfect. For example, since the number of training samples is not sufficient to train a deep network for learning better invariant features, a better preprocessing method that can reduce finger vein rotation and displacement may improve the system.

### 1.6. Contribution

In summary, vein pattern-based methods and feature point matching methods need to segment the vein pattern and are affected by the image quality. Most of the methods based on the principle of statistical feature analysis ignore the local detail information of the image, and the DL approach relies on a large dataset and computational power. In addition, the finger vein recognition methods based on local binary features mostly use manually designed local features, which have weak differentiation ability and cannot reflect the essential features of the data. Moreover, the dimensionality is too high, the algorithm is complicated, and the processing speed is slow.

**Figure 1:**
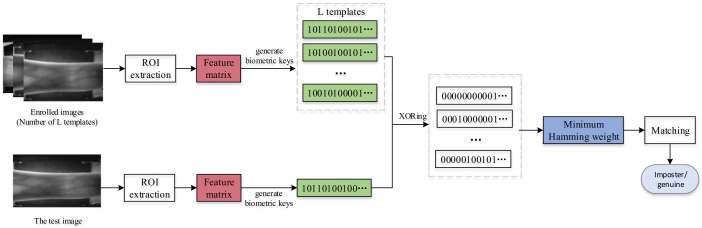
Overall framework of the proposed method.

To solve the LBP dimensionality problem, inspired by the work of MB-LBP and CS-LBP, we propose a new operator BACS-LBP to encode images for recognition, as shown in Figure 1. Our main contributions are as follows:

1. CS-LBP analyzes texture features from a microscopic perspective, which will ignore large texture structures by direct use for finger vein recognition, and the block mean matrix in [36] emphasizes the local macro features. Therefore, we add the block averaging idea to the CS-LBP, which can take into account local macro and micro information and make up for the shortage of CS-LBP. The experimental results show that our method has good recognition rate.
2. Our method combines local macroscopic features and microscopic features, which can describe image features more comprehensively. Moreover, the characteristic dimension of BACS-LBP is only 1/8 of that of LBP. Consequently, our method has less dimension and less data redundancy.
3. Our method is computationally simple, with no need for segmentation of the image and complex preprocessing process, so we reduce the time cost compared to traditional methods.
4. In the matching process, the minimum Hamming distance is used for matching: save multiple templates instead of one during registration, compare the samples with all templates during verification, and take the ratio of the minimum value to the codeword length as the matching score.

The rest of the paper is organized as follows. In Section 2, we discuss the proposed method. Experimental results are presented and the analysis of our approach is presented in Section 3. Finally, Section 4 concludes the paper.

## 2. Proposed Approach

In this section, our proposed approach is discussed concretely, and Figure 1 shows the overview of the proposed approach. The following sections detail the different tasks involved in our approach.

### 2.1. Calculating the Matrix after Blocking and Averaging

The first step of BACS-LBP needs to calculate the block and average matrix. To achieve this, we first divide the finger vein image into a certain number of blocks. The local features of each region of the image usually differ greatly. If the entire image is processed directly, the local differences information will be lost. The block method can enhance the robustness of the image to noise and improve the coarse-grained grasp of the overall information. Theoretically, smaller and more refined blocks can bring better local description capabilities, but they can also produce higher-dimensional composite features, increasing the time complexity of calculations, and too-small blocks will lose statistical significance, cause overfitting phenomenon, and reduce the recognition rate. In this paper, the optimal number of blocks is selected in the experiment to balance the contradiction between the recognition time and the recognition accuracy, which is described in detail in the experimental analysis.

The block method is as follows: let I be the finger vein image, and then divide I into a number of small blocks each of size a×b pixels, where a,b≪A,B, A×B being the size of I. Specifically, I can be denoted as a p×q matrix of all blocks, as follows.

(1) $$\begin{array}{c} I=\left(\begin{array}{cccc} I_{11} & I_{12} & \cdots & I_{1q}\\ I_{21} & I_{22} & \cdots & I_{2q}\\ \vdots & \vdots & \ddots & \vdots \\ I_{p1} & I_{p2} & \cdots & I_{pq} \end{array}\right), \end{array}$$

where $I_{ij}$ is the (i,j)-block of I. Note that the blocks, which are on the boundary, may not be of equal size, so we add element 0 to make it equal.

**Figure 2:**
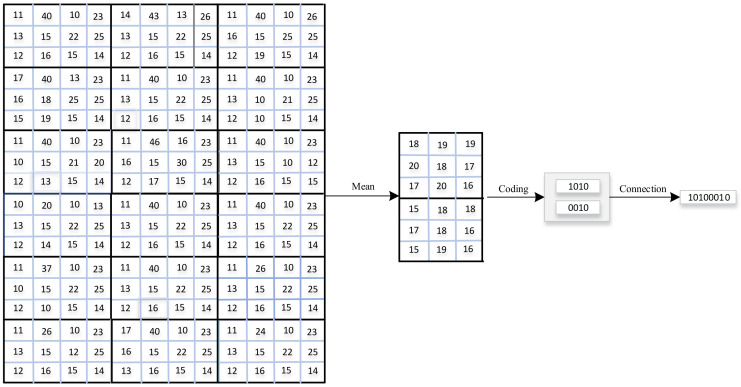
Process of BACS-LBP algorithm.

After dividing into blocks, we will calculate the average value of each small block, and the resulting matrix will be used as the input part of the encoding. The whole process of calculating the block and average matrix is shown in Figure 2.

### 2.2. Generating Code

**Figure 3:**
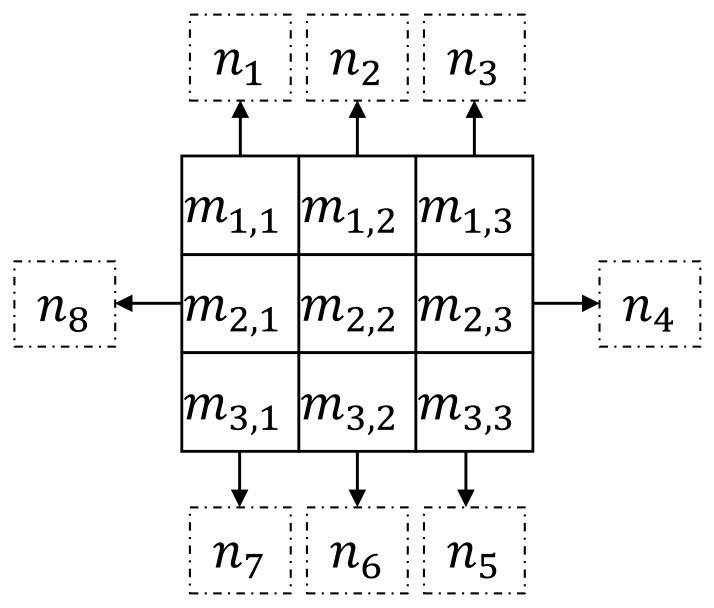
Example of matrix ${\left[m_{i,j}\right]}_{i,j=1}^{3}$ to $n_{j}$.

The second step of the BACS-LBP algorithm is to carry out centrosymmetric coding. Specifically, we divide the matrix obtained from the appeal into 3×3 small matrices (0 is used to supplement when the boundary is insufficient). The idea of CS-LBP encoding is adopted, that is, the small matrix is encoded according to Equation (2) to obtain the binary sequence $x_{i}$. All binary sequences $x_{i}$ are connected to form the vein codeword X. The process can be expressed by the following Equation (3) or Figure 2:

(2) $$\begin{array}{cc} x_{i,j} & =\left\{\begin{array}{cc} 1, & n_{j}\geq n_{j+4};\\ 0, & n_{j}<n_{j+4}. \end{array}\right. \end{array}$$

(3) $$\begin{array}{cc} x & =\left\{x_{1}\left|\right|x_{2}\left||\cdots |\right|x_{m}\right\}, \end{array}$$

where j=1, 2, 3, 4, $x_{i,j}$ is the *j*-th element in $x_{i}$, and $x_{i}$ is the binary sequence of each small matrix, and x is the binary code generated by the whole graph. The pixel value of a point, in the field of any point in the image, is marked as $n_{j},\;j=1,2,\cdots ,8$. In Figure 3, we show how $n_{j}$ is obtained from the small matrix ${\left[m_{i,j}\right]}_{i,j=1}^{3}$.

In the process of obtaining the biological feature binary codes through appeal, we only used simple “comparison” and “connection” operations without complicated calculations. Therefore, the proposed method has low computational complexity and faster coding speed.

### 2.3. From Code to Matching

The minimum Hamming distance was proposed to judge the similarity of the enrolled code and the sample code. The proposed method is different from other methods that use Hamming distance matching. In the registration stage, we select binary codes of *N* images for registration as template, i.e., there are multiple templates ${\left\{x^{\left(n\right)}\right\}}_{n=1}^{N}$ saved instead of one, where $x^{\left(n\right)}$ is obtained from the *n*-th image by Equation (3). In the matching stage, we calculate the Hamming distance between the sample code and all the enrolled codes, and take the minimum Hamming distance value. The ratio of this value to the length of the codeword is taken as the matching score between the sample code and all the enrolled subjects. This method of measuring similarity can reduce the intra-instance distance and increase the inter-instance distance, thereby increasing the recognition rate.

Concretely, the matching score definition of the proposed method and the entire encoding process can be summarized by Algorithm 1, and ⊕ denotes the XOR operator, which is used to highlight the differences and similarities between two binary sequences.
**Algorithm 1** The calculation of the proposed method.**Input**: Image I**Output**: The matching score (Smatching) 1: I=Blocking(I) 2: **for** i=1→p **do** 3:    **for** j=1→q **do** 4:      $I^{\prime }\left(i,j\right)=\mathrm{mean}\left(I_{ij}\right)$ 5:    **end for** 6: **end for** 7: Divide $I^{\prime }$ into *m* blocks of 3×3 matrix, i.e $I_{1}^{\prime },...,I_{m}^{\prime }$ 8: Calculate $x_{i}=\mathrm{CS}-\mathrm{LBP}\left(I_{i}^{\prime }\right)$ 9: Set $x=(x_{1}||x_{2}||\cdots ||x_{m})$ 10: The enrolled binary codes: $\left\{x^{\left(n\right)}:n=1,2,\cdots ,N\right\}$ 11: **for** n=1→N **do** 12:    $S_{n}=\mathrm{sum}\left(x^{\left(n\right)}⊕x\right)$ 13: **end for** 14: $\mathrm{Smatching}=\frac{\min_{n}S_{n}}{\mathrm{Length}(x)}$

## 3. Experiments and Experimental Results

### 3.1. Databases

Two open finger vein databases are used to evaluate the performance of our proposed approach. As some databases only include six images of each finger, we ignore the fingers with six images and only use the fingers with 12 images to ensure the consistency of all databases. The details of these databases are given below.

(1) HKPU Database [12]: The available database of Hong Kong Polytechnic University (HKPU) has 3132 images from 312 fingers; all the images are in bitmap format with a resolution of 513×256 pixels. The finger images were acquired in two separate sessions. Each of the fingers provided six image samples in each session, resulting in a total of 12 images of finger obtained, but in the database version we used, only the first 210 fingers had 12 pictures, and others each has six images.
(2) USM Database [45]: The database of Universiti Sains Malaysia (USM) consists of 492 fingers, and every finger provided 12 images. The spatial resolution of the captured finger images was 640×480. This database also provides the extracted ROI images for finger vein recognition using their proposed algorithm described in [45].

Table 1 gives the detailed information of the two databases we used.

### 3.2. Experimental Protocols

Two experiments are designed here. In the first one, we evaluated the parameters (i.e., the template number and decision threshold (DT)) that affect the recognition performance, and selected the most appropriate value for the following experiments. In the second experiment, we compared the proposed method with some typical recognition methods and some state-of-the-art finger vein recognition methods to prove the potential of our method in the recognition task.

The database setup of the recognition task in the experiment is given. For the open database HKPU, we only consider the fingers with 12 pictures. There are 210 finger samples in total. The USM database has 12 images of all fingers, and the images in the whole database are used for the experiment. It is observed that values of the size of the block matrix have a very small impact on the performance of the algorithms, and there are differences in image size between the two databases. Therefore, the effect of block size on the recognition rate is not listed in the result. In the experiment, we select the appropriate block size directly; for HKPU and USM data, the block size is 3×8 and 5×5, respectively. Equal error rate (EER) and recognition rate are employed to assess recognition performance. The EER means the value whereby the false acceptance rate (FAR) is equal to the false rejection rate (FRR). We use the intra-instance (1:1) and inter-instance (1:*N*) as the main indicators to measure the test identification.

### 3.3. Selection of Parameter Value

**Figure 4:**
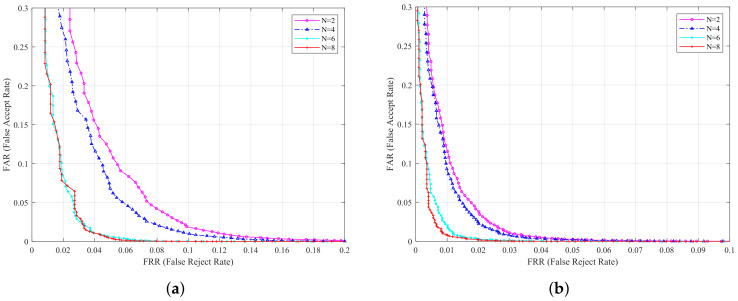
ROC curves with different templates on two databases: (**a**) on HKPU database; (**b**) on USM database.

In this experiment, we discuss the performance of the proposed method with distinct parameter values, which contain the number of template and decision threshold (DT). This experiment is implemented on the most popular HKPU database and USM database. We fix the DT when considering the influence of different template numbers on the recognition rate, and vary the template number from 2 to 8, with 2 as the interval. When the number of templates is *N*, the first *N* images of the database are selected as the template, and the remaining images are used as the test samples. The recognition performance are listed in Table 2, and Figure 4 shows the ROC curves of two databases under different templates.

It can be distinctly found from the above results that the recognition performance enhances by increasing the number of templates. Plainly, larger number of templates can more effectively extract details of finger vein patterns, which contributes to recognition performance. Furthermore, the results also show that, when the number of templates varies from six to eight, to improve recognition performance is not significant. Considering the convenience of image collection in practical application, we use six templates in following experiments.

Next, we fix the template number at six and change the number of matching score from 0.18 to 0.21, with 0.01 as the interval to obtain the DT. The recognition rate of DT under different values is presented in Table 3.

From the results, we can see that there is a critical DT value. If this threshold value is exceeded, the 1:1 recognition rate rises and the 1:*n* recognition rate decreases. This threshold will vary with the change of finger vein database, which means that different finger vein databases need to adjust the corresponding threshold to obtain the best recognition accuracy. For clarity, we use the equal error rate (ERR) indicator to compare the different performances.

### 3.4. Impact of Block Size on Performance

**Figure 5:**
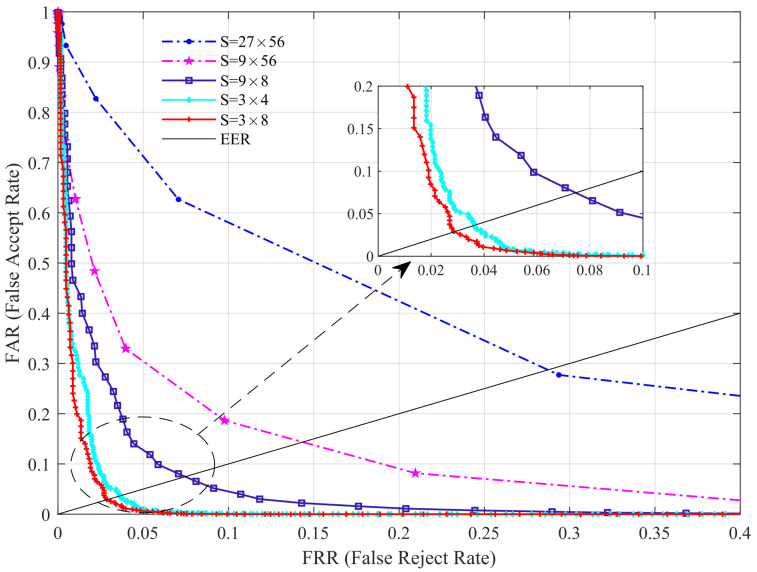
Performance comparison under different block size.

In our previous study [30], we found that for a given image size, the higher the number of blocks, the higher the recognition rate, and when the number of blocks reaches a certain number, the recognition rate does not decrease. Based on this, we mainly explore the effect of the number of blocks on the performance of the proposed scheme in this section. Here, we conduct experiments on the HKPU database. First, we fix the number of templates to six and vary the block size *S* to investigate the EER values under different *S* (S=3×4,3×8,9×8,9×56,27×56). The results are shown in Table 4 (Figure 5).

From Table 4, it can be observed that the EER is slowly getting smaller as the number of blocks increases, i.e., the *S* gradually decreases, but when the S decreases from 3×8 to 3×4, the EER shows an increasing trend again. This can be explained by the fact that a larger number of blocks leads to insufficient texture information between different images, and a smaller number causes local noise, which leads to a lower recognition rate. Therefore, on the HKPU database, we set the *S* to 3×8. The selected block size naturally differs due to the differences in image sizes between databases. Our experiments on the USM database show that a block size of 5×5 is the most suitable, and is not listed here.

### 3.5. Evaluation of BACS-LBP

**Figure 6:**
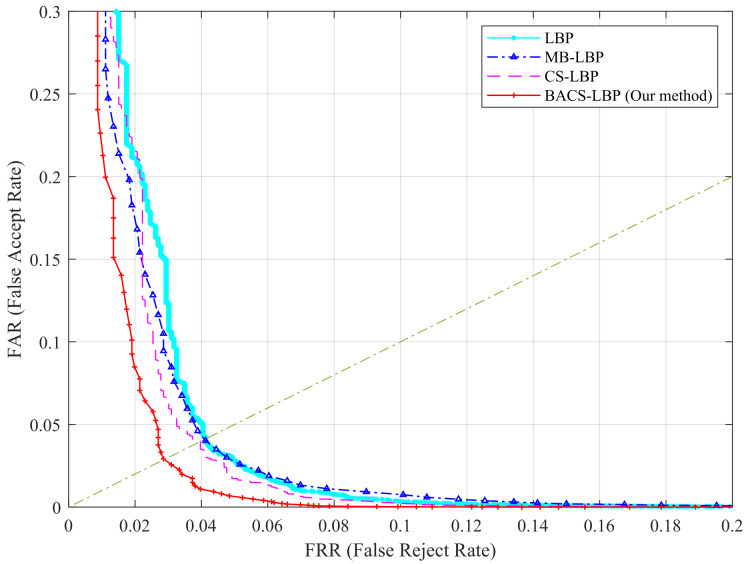
Performance comparison of LBP, MB-LBP, CS-LBP, and BACS-LBP.

In this section, to verify the performance of the proposed scheme, we test LBP, MB-LBP, CS-LBP, and BA-CSLBP operator on the HKPU database with the same parameters. The results are shown in Figure 6, and it can be seen that our operator exhibits better performance. This is because our operator takes into account both macroscopic and microscopic local information and has a better representation of the image.

In addition, the time we spend to extract a sequence of images and the time consumed by the proposed scheme to match each image with other schemes are given in Table 5. The numerical results of the experiments show that our method is simpler and less computationally intensive, which also means that it is well suited for embedded and mobile systems.

**Figure 7:**
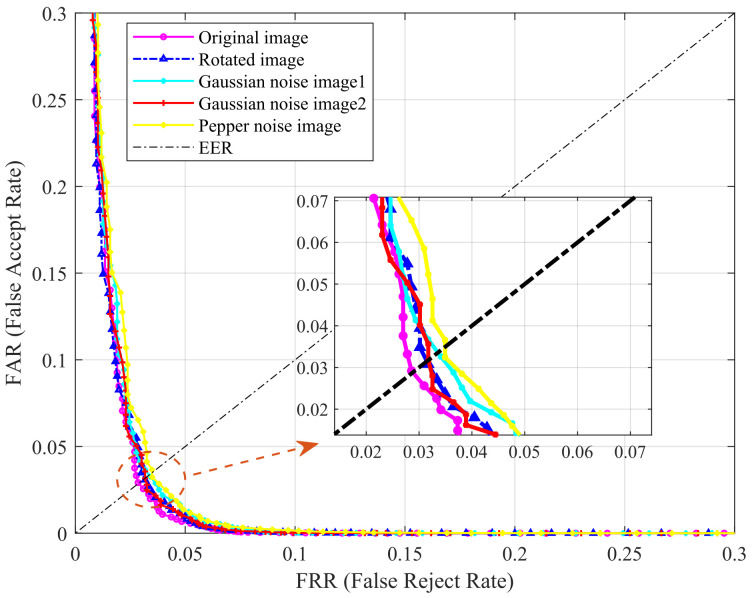
Robustness testing.

Furthermore, we designed relevant experiments to test the sensitivity of the method, i.e., rotate the image or add noise, and compare the performance with the original image. The tests were performed on the HKPU database, as shown in Figure 7. We added a total of two types of noise, i.e., Gaussian noise and pepper noise. “Gaussian noise image1” and “Gaussian noise image2” in Figure 7 indicate the addition of Gaussian noise at a signal-to-noise ratio of 30 dB and 40 dB, respectively. The results in the figure clearly show that adding noise and rotation have little effect on the performance, which further confirms the robustness of the proposed method.

### 3.6. Compared with Previous Work

In this section, we examined the performance of the proposed method in recognition mode by comparing with the various existing finger vein recognition methods. The comparison is performed on HKPU databases and USM databases. The performance is reflected by the EER. Table 6 shows the EER of different methods on HKPU databases. Specific analysis of these results is described below.

First, our proposed method is compared with the algorithm without segmentation (e.g., LBP [28], ELBP [16], MB-LBP [36], ${\left(2D\right)}^{2}$ PCA [22], BMSU-LBP [27], CS-LBP [37]). We can see that our method has better recognition performance. This is because these methods only consider the local information of the image. Compared with these methods, our method is able to represent the image information more completely, is robust to small local variations, and has stronger noise immunity.

Second, in comparison with the algorithm with segmentation (e.g., RLT [8], MC [10], MCP [9], ASAVE [2], WVI [13], etc.), our proposed approach achieves better performance on the HKPU databases. The underlying reasons are that some images in the database have limited vein patterns, low contrast between vein and non-vein regions, and segmentation algorithms are mostly sensitive to environmental changes such as illumination and finger pose. However, our algorithm does not need to segment them, first capturing the overall information by block averaging, and then further refining the local features with CS-LBP algorithm. Thus, to some extent, we are more tolerant to environmental factors.

Finally, there are some methods that have used the CNN models in recent years. Because the CNN network is difficult to train, as per the original paper, it has a high possibility that the result will be worse than the original paper. On the other hand, it needs a large training set and large numbers of data to be labeled. Therefore, our data directly cites the results of the original paper (some papers only give the correct identification rate (CIR)). The results are shown in Table 7.

From the table, it can be seen that our method is still comparable to some of the CNN-based methods. For instance, the CIR of the proposed method on two databases is greater than 98%, but the CIR in [40] on both databases is less than 98%. Of course, we can still see that some of the CNN-based methods have achieve better results than the proposed method. The CNN-based methods may have good results when a good network is training, but it needs careful parameter fine-tuning. Moreover, the finger vein images are very different to the natural images in terms of image qualities. Hence, much work should be carried out to improve the CNN-based finger vein recognition methods.

## 4. Conclusions and Future Work

Existing finger vein recognition methods are not satisfactory regarding the recognition performance. Algorithms that need to segment images (such as maximum curvature, repeated linear tracking, Gabor filtering, ASAVE, etc.) have high requirements for image quality and are not practical. The method without segmentation algorithm (such as LBP) is computationally complex and has large data redundancy, so it is not effective to directly use it in finger vein recognition. The proposed finger vein code generation algorithm is simple in calculation, does not need complex segmentation algorithm, can overcome the problem of low image quality, and has stronger robustness to image noise.

The extensive experiments on two finger vein databases were conducted for verifying the effectiveness of the proposed method. From the experimental results, we can obviously see that the proposed method outperforms most of the latest methods and has competitive potential in finger vein recognition. In future work, it is hoped that finger vein databases can be collected on a large scale: because there is no public large database to compare the performance of finger vein recognition methods, the image differences in each database will not be conducive to the fair evaluation of recognition performance.

## Figures and Tables

**Table 1:** The details of databases.

DB	Finger Number	Number per Finger	Size of Raw Image	ROI Image
HKPU	312	6/12	513×256	Using the method of [12]
USM	492	12	640×480	From DB

**Table 2:** The recognition performance under different number of templates.

DB	The Template Number	2	4	6	8
HKPU	EER (%)	6.88	5.52	2.86	2.91
USM	EER (%)	2.35	2.08	1.16	0.92

**Table 3:** The recognition rate under different DT values.

DB	The Decision Threshold	Intra-Instance (1:1)			Inter-Instance (1:*n*)		
	**DT Value**	**Total** **Times**	**False** **Times**	**Recognition** **Rate** **(%)**	**Total** **Times**	**False** **Times**	**Recognition** **Rate (%)**
HKPU	0.18	1260	55	95.6	263,340	2465	99.1
	0.19	1260	43	96.6	263,340	5228	99.0
	0.20	1260	34	97.3	263,340	9899	96.2
	0.21	1260	29	97.7	263,340	16911	93.6
USM	0.18	2952	83	97.1	1,449,432	2000	99.7
	0.19	2952	57	98.1	1,449,432	5127	99.6
	0.20	2952	39	98.7	1,449,432	11263	99.2
	0.21	2952	32	98.9	1,449,432	20730	98.7

**Table 4:** The recognition performance under different block size.

*S*	27×56	9×56	9×8	3×8	3×4
EER (%)	28.54	14.21	7.54	2.86	3.73

**Table 5:** Time comparison.

Methods	Feature Extraction	Matching Time per Image	DB
ASAVE [2]	19.7 s	65.7 ms	HKPU
CPBFL-BCL [3]	-	32.5 ms	USM
Wide Line Deterctor [7]	17.9 s	19.5 ms	HKPU
This paper	13.4 s	3.6 ms	HKPU
	15.1 s	3.7 ms	USM

**Table 6:** Comparison of the recognition performance of our proposed method and existing methods in two databases.

DB	Method	Algorithm	EER (%)
HKPU	No segmentation	LBP [28]	4.2
		MB-LBP [36]	4.1
		ELBP [16]	5.59 *
		CS-LBP [37]	3.97
		${\left(2D\right)}^{2}$ PCA [22]	3.57
	Need segmentation	RLT [8]	16.31 *
		MC [10]	4.03
		MCP [9]	18.99 *
		CRS [11]	2.96
		Gabor [12]	4.61 *
		ASAVE [2]	2.91 *
		WVI [13]	3.33 *
		This paper (BACS-LBP)	2.86
USM	No segmentation	BMSU-LBP [27]	1.89 **
		CS-LBP [37]	6.06
		This paper (BACS-LBP)	1.16

* Cited from [13]; ** Cited from [27].

**Table 7:** Comparison between the proposed method and deep learning method.

REF	Method	DB	Performance (%)
[1]	CNN	HKPU	EER=2.70
		USM	EER=1.42
[38]	CNN with Triplet similarity loss	HKPU	EER=13.16
	Supervised discrete hashing with CNN	HKPU	EER=9.77
[39]	CNN	HKPU	EER=2.33
		USM	EER=0.80
[40]	CNN with original images	HKPU	CIR=95.32
		USM	CIR=97.53
	CNN with CLAHE enhanced images	HKPU	CIR=94.37
		USM	CIR=97.05
	Our proposed method (BACS-LBP)	HKPU	EER=2.86
			CIR=98.8
		USM	EER=1.42
			CIR=98.8

## Data Availability

The HKPU database and the USM database used in this paper can be downloaded from http://www4.comp.polyu.edu.hk/~csajaykr/fvdatabase.htm, http://drfendi.com/fv_usm_database/ respectively, accessed on 10 March 2022.
